# Copper mediates auxin signalling to control cell differentiation in the copper moss *Scopelophila cataractae*

**DOI:** 10.1093/jxb/eru470

**Published:** 2014-11-26

**Authors:** Toshihisa Nomura, Misao Itouga, Mikiko Kojima, Yukari Kato, Hitoshi Sakakibara, Seiichiro Hasezawa

**Affiliations:** ^1^RIKEN Center for Sustainable Resource Science, 1-7-22 Suehiro-cho, Tsurumi, Yokohama, Kanagawa 230-0045, Japan; ^2^Department of Integrated Biosciences, Graduate School of Frontier Sciences, The University of Tokyo, 5-1-5 Kashiwanoha, Kashiwa, Chiba 277-8562, Japan

**Keywords:** Auxin, bryophytes, cell differentiation, copper, copper mosses, heavy metal, metallophyte.

## Abstract

The copper (Cu) moss *Scopelophila cataractae* (Mitt.) Broth. is often found in Cu-enriched environments, but it cannot flourish under normal conditions in nature. Excess Cu is toxic to almost all plants, and therefore how this moss species thrives in regions with high Cu concentration remains unknown. In this study, we investigated the effect of Cu on gemma germination and protonemal development in *S. cataractae*. A high concentration of Cu (up to 800 µM) did not affect gemma germination. In the protonemal stage, a low concentration of Cu promoted protonemal gemma formation, which is the main strategy adopted by *S. cataractae* to expand its habitat to new locations. Cu-rich conditions promoted auxin accumulation and induced differentiation of chloronema into caulonema cells, whereas it repressed protonemal gemma formation. Under low-Cu conditions, auxin treatment mimicked the effects of high-Cu conditions. Furthermore, Cu-induced caulonema differentiation was severely inhibited in the presence of the auxin antagonist α-(phenylethyl-2-one)-indole-3-acetic acid, or the auxin biosynthesis inhibitor l-kynurenine. These results suggest that *S. cataractae* flourishes in Cu-rich environments via auxin-regulated cell differentiation. The copper moss might have acquired this mechanism during the evolutionary process to benefit from its advantageous Cu-tolerance ability.

## Summary

The habitat of copper moss— *Scopelophila cataractae*—is restricted to Cu-enriched environments. We show that the early stage of protonemal development in *S. cataractae* is controlled by Cu through auxin signalling.

## Introduction

Copper (Cu) is an essential micronutrient for normal plant growth and development. Plants use Cu for many physiological processes such as photosynthesis, respiration, protection against oxidative stress, cell wall lignification, and ethylene perception (Festa and Thiele, 2011; Marschner and Marschner, 2012; Palmer and Guerinot, 2009). However, Cu is also one of the most toxic heavy metals, and excess Cu concentrations induce oxidative stress via the Haber–Weiss and Fenton reactions (Halliwell and Gutteridge, 1984), which affect various enzymatic activities and biological processes (Sudo *et al.*, 2008; Van Assche and Clijsters, 1990; Yuan *et al.*, 2013). Although normal plants cannot healthily grow in Cu-polluted sites such as around copper mines or artificial copper products, some bryophytes, called ‘copper mosses’ (Persson, 1956; Shaw, 1994) are occasionally found in such environments. Copper mosses are metallophytes that are tolerant to high concentrations of heavy metals. They may be categorized into two types, ‘obligate metallophytes,’ which are only found in the presence of metals, and ‘facultative metallophytes,’ which are tolerant to such conditions but are not confined to them.

A typical copper moss *Scopelophila cataractae* (Mitt.) Broth. is distributed worldwide in Cu-rich environments (Shaw, 1987). In Asian countries, *S. cataractae* colonies are often found under the copper roofs of Buddhist temples and shrines and around copper mines (Sakurai, 1934; Satake *et al.*, 1988; Shaw, 1987). This moss is highly tolerant to Cu and accumulates large amounts of Cu in its plant body (Aikawa *et al.*, 1999; Konno *et al.*, 2010; Nomura and Hasezawa, 2011; Satake *et al.*, 1988). Indeed, it requires a Cu-rich environment for optimal growth (Shaw and Owens, 1995) and is thought to be an obligate metallophyte because its habitat is severely restricted to Cu-enriched environments. The average Cu content of the habitat substrate for *S. cataractae* has been reported to be 7.1±6.1g kg^−1^ dry weight soil (Aikawa *et al.*, 1999). On the other hand, the reported maximum Cu content of *S. cataractae* is approximately 3% in dry weight (Satake *et al.*, 1988). Therefore, this moss species may be defined as a hyperaccumulator (Palmer and Guerinot, 2009; Rascio and Navari-Izzo, 2011).

Bryophytes expand their habitat into new locations through two major mechanisms: formation of spores and gemmae. After the spores or gemmae germinate, the emerging protonema cells (called chloronema) propagate, while the apical cells differentiate into another type of protonema cells (called caulonema), which have spindle-shaped chloroplasts and oblique septa (Cove *et al.*, 2006). In most mosses, the caulonema cells develop from caulonemal side-branch initial cells that can differentiate into secondary chloronemal apical cells or buds. Each bud grows and finally forms a leafy gametophore (Aoyama *et al.*, 2012; Cove *et al.*, 2006; Cove and Knight, 1993). During the developmental process, the differentiation of chloronema to caulonema is influenced by environmental conditions such as nutrition and light, and it is usually promoted when species-specific conditions are optimal (http://www.bryoecol.mtu.edu/). The phytohormone auxin has been reported to be involved in the mechanism underlying this differentiation; exogenous auxin treatment is known to induce differentiation of chloronema to caulonema in *Physcomitrella patens* and *Funaria hygrometrica* (Ashton *et al.*, 1979; Cove *et al.*, 2006; Johri and Desai, 1973; Thelander *et al.*, 2005). In addition, auxin positively regulates the expression of the ROOT HAIR DEFECTIVE SIX-LIKE1 (PpRSL1) and PpRSL2 basic helix–loop–helix (bHLH) transcription factors, and their overexpression promotes the differentiation of chloronema to caulonema in *P. patens* (Jang and Dolan, 2011). A recent study revealed that auxin induces the expression of AP2-type transcription factors orthologous to *Arabidopsis thaliana* AINTEGUMENTA, PLETHORA, and BABY BOOM (APB), which are essential for the cytokinin-dependent induction of gametophore apical cells in *P. patens* (Aoyama *et al.*, 2012).

On the other hand, some mosses, including *S. cataractae*, can form gemma at their chloronema tips in the protonemal stage (http://www.bryoecol.mtu.edu/; Nomura and Hasezawa, 2011; Rumsey and Newton, 1989). Protonemal gemma formation has been speculated to allow rapid escape from a hostile environment (http://www.bryoecol.mtu.edu/). Sporophyte formation in *S. cataractae* is very rare, and asexual reproduction by gemma formation is considered the main mechanism used by this species to expand its habitat into new locations (Rumsey and Newton, 1989; Shaw, 1994). Our previous study revealed that the frequency of protonemal gemma formation is affected by the environmental Cu concentration of the medium used to grow *S. cataractae*; gemma formation occurred more frequently under low Cu concentrations (Nomura and Hasezawa, 2011). However, the regulatory mechanism underlying cell differentiation from chloronema to caulonema or gemma in response to environmental factors is still unclear.

In this study, we investigated the effect of Cu on the differentiation efficiency of *S. cataractae* from chloronema to caulonema or protonemal gemma to understand why the growth of this moss species is restricted to Cu-rich environments. Our results suggest that cell differentiation from the chloronema is regulated by the environmental Cu concentration via auxin signalling. The unique Cu-regulated auxin accumulation and cell differentiation system might explain the exclusive distribution of *S. cataractae* in Cu-rich environments.

## Materials and methods

### Measurement of soluble copper content in the habitat soil of *S. cataractae*

To estimate the soluble copper concentration in the habitat of *S. cataractae*, we collected the substrate soil of *S. cataractae* from the Zenpukuji Temple in the Ibaraki Prefecture, which is the place of origin of the ScZEN culture strain. Each soil sample (points 1–5) was collected at intervals of at least 50cm. We also collected a soil sample from the Tsurumi Shrine in the Kanagawa Prefecture, which is another habitat of *S. cataractae*. Soil extract solutions were prepared by centrifugation at 14,000rpm for 5min using a centrifuge spin column (Ultrafree-MC, 0.1-µm pore size, Merck Millipore, Darmstadt, Germany). The Cu concentration was determined by inductively coupled plasma mass spectrometry (ICP-MS; NexION300, Perkin Elmer) after diluting the sample with HCl (0.01mol l^–1^). The moisture content of the soil was calculated by subtracting the dry (200 °C, 2h) soil weight from the fresh soil weight.

### Plant materials and growth conditions

*S. cataractae* protonemal cell cultures were established as described previously (Nomura and Hasezawa, 2011). This culture strain of *S. cataractae*, which was originally collected from the Buddhist temple Zenpukuji, Ibaraki Prefecture in Japan, was named ScZEN. Protonemal cells were cultured in BCDAT liquid medium (Nishiyama *et al.*, 2000) containing 0.2% sucrose with shaking (140rpm) at 23 °C under conditions of 16-h light/8-h dark photoperiod at an intensity of 50 µmol photons m^–2^·s^–1^. The basal Cu concentration of BCDAT medium is 0.22 µM.

### Collection and culture of *S. cataractae* gemmae

When *S. cataractae* protonemal cells were cultured in BCDAT liquid medium, the gemmae adhered to and accumulated on the inner wall of the glass bottle (Supplementary Fig. S1A). The collected gemmae were resuspended in fresh BCDAT liquid medium and then spread on BCDAT agar medium using an autoclaved paintbrush (Supplementary Fig. S1B, C). The gemmae on the medium were cultured at 23 °C under conditions of 16-h light/8-h dark photoperiod at an intensity of 50 µmol photons m^–2^·s^–1^ (Supplementary Fig. S1C, D).

### Treatment with heavy metal, auxin, cytokinin, and other reagents

Cu treatment was performed by culturing the gemmae on BCDAT agar medium supplemented with Cu in the form of CuSO_4_, Cu(NO_3_)_2_, or CuCl_2_. Co-treatment with ethylenediaminetetraacetic acid (EDTA) and Cu was performed by culturing the gemmae on BCDAT agar medium containing 400 µM EDTA and 400 µM CuSO_4_. According to the chemical speciation program Geochem-EZ (Shaff *et al.*, 2009), 99% of Cu ions are chelated with EDTA under these conditions. Other heavy metals were supplemented in the form of 400 µM MnSO_4_, CoSO_4_, NiSO_4_, or ZnSO_4_. Treatment with auxins, the auxin antagonist α-(phenylethyl-2-one)-indole-3-acetic acid [PEO-IAA; stock solutions made in dimethyl sulfoxide (DMSO) to 20 mM] (Hayashi, 2012; Hayashi *et al.*, 2012), or l-kynurenine (stock solutions prepared in 50% DMSO solution diluted in sterile distilled water to 40mM; Sigma-Aldrich) were performed by culturing the gemmae on BCDAT agar medium supplemented with auxin in the form of 0.5 µM indole acetic acid (IAA) or naphthalene acetic acid (NAA), 20 µM PEO-IAA, or with 20 and 40 µM l-kynurenine. Cytokinin treatment was performed by culturing the gemmae on BCDAT agar medium supplemented with 0.5 or 2 µM 6-benzylaminopurine (BAP).

### Microscopic observation of germinating gemmae and protonema development on agar medium

Images of gemmae cultured on BCDAT agar medium for 10 d were obtained using a CCD camera attached to a stereomicroscope (SZX12; Olympus, Tokyo, Japan). To determine the population percentages of caulonema cells and protonemal gemmae, gemmae cultured for 10 d under various conditions were collected on a slide glass, and images of the germinating gemmae were obtained using a CCD camera attached to a microscope (BX51, Olympus). The population percentages of caulonema cells and gemmae were quantified from these pictures.

### Measurement of endogenous levels of IAA and other phytohormones

Gemmae were cultured on BCDAT agar medium containing 0.22 (control), 400, or 800 µM CuSO_4_, or 400 µM CuSO_4_ with 400 µM EDTA. Plates were covered with cellophane, cultured for 10 d, and plant samples were collected using cell scrapers. The samples (approximately 50mg fresh weight) were frozen in liquid nitrogen. Phytohormone extraction and identification were performed by ultra-performance liquid chromatography (UPLC)-tandem mass spectrometry (AQUITY UPLC System/XEVO-TQS; Waters) using an ODS column (AQUITY UPLC BEH C18, 1.7 µm, 2.1×100mm; Waters, Milford, MA, USA), as described previously (Kojima *et al.*, 2009).

## Results

### Effects of Cu on germination and differentiation

**Fig. 1.**
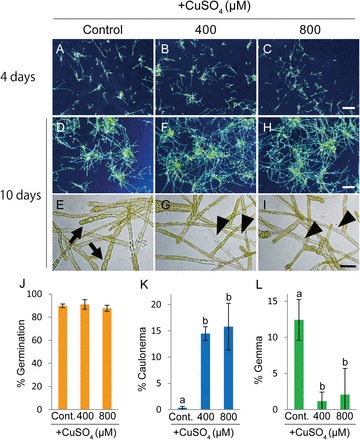
Effects of copper on gemma germination and chloronema cell differentiation in *Scopelophila cataractae*. Gemmae were germinated on BCDAT agar media containing 0.22 (control), 400, or 800 µM CuSO_4_. Photographs of growing protonema were obtained after incubation for 4 (A–C) or 10 d (D–I). E, G, and I are magnified images of protonemal cells from D, F, and H, respectively. The arrows in E indicate gemmae formed at the protonema tips. The arrowheads in G and I indicate caulonema cells. Scale bars=1mm for A–D, F, and H; and 50 µm for E, G, and I. (J) Germination rate after 4 d of culture on agar media containing 0.22 (control), 400, or 800 µM CuSO_4_. Population percentages of caulonema cells (K) and gemma (L) after 10 d of culture on agar media. Values represent the means±standard deviation (SD) of four independent experiments. Different letters indicate statistically significant differences detected by Tukey–Kramer tests (*P*<0.05) following one-way analysis of variance (ANOVA).

In this study, we used gemmae as our starting culture material, because *S. cataractae* almost always reproduces asexually by gemma formation. To investigate whether gemma germination is affected by the surrounding Cu concentration, we first determined the effect of treatment with various Cu concentrations on gemma germination. High Cu concentrations (400 and 800 µM) had no effect on the gemma germination rate of *S. cataractae* (Fig. 1A–C, J). Analysis of soil extract solutions revealed that the soluble Cu concentration of the habitat of *S. cataractae* ranged from 160–1700 µM (Supplemental Table 1), indicating that our experimental condition is within the range for *S. cataractae* habitats.

We next investigated the effect of Cu on protonema differentiation in *S. cataractae*. Treatment with 400 and 800 µM CuSO_4_ promoted transition from chloronema to caulonema (Fig. 1F–I, K, Supplementary Fig. S2A, C) but repressed protonemal gemma formation (Fig. 1L, Supplementary Fig. S2B). After treatment with 800 µM CuSO_4_, the population percentage of caulonema increased from 0.6% to 15.8% (Fig. 1K), whereas protonemal gemma formation decreased from 12% to 2% (Fig. 1L). In the control condition (0.22 µM Cu), protonema growth was repressed compared with that in the Cu-treated condition (Fig. 1D, F, and H) because tip growth was arrested by protonemal gemma formation (Fig. 1E). A Cu concentration of 200 µM was found to be sufficiently toxic to severely inhibit protonemal growth in *P. patens* (Nomura and Hasezawa, 2011).

**Fig. 2.**
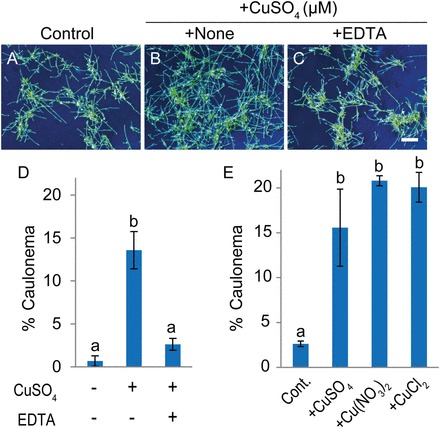
Effects of co-application of ethylene diaminetetraacetic acid (EDTA) with copper on the differentiation of chloronema to caulonema in *Scopelophila cataractae*. Gemmae were germinated on BCDAT agar media containing 0.22 µM CuSO_4_ (control, A), 400 µM CuSO_4_ (B), and 400 µM CuSO_4_ and 400 µM EDTA (C). After 10 d of incubation, photographs of growing protonema were obtained. Scale bar=1mm. (D) Population percentage of caulonema cells was determined after 10 d of culture on agar media. Values represent the means±SD of three independent experiments. (E) Gemmae were germinated on BCDAT agar media containing 0.22 µM CuSO_4_ (control), 400 µM CuSO_4_, 400 µM Cu(NO_3_)_2_, or 400 µM CuCl_2_. After 10 d of incubation, the population percentages of caulonema cells were quantified. Values represent the means±SD of three independent experiments. Different letters indicate statistically significant differences as detected by Tukey–Kramer tests (*P*<0.05) following ANOVA.

When *S. cataractae* protonemata were grown in the presence of both Cu and the metal-chelating reagent EDTA, the Cu-induced differentiation of chloronema to caulonema was significantly repressed (Fig. 2A–D). In addition, treatment with Cu(NO_3_)_2_ or CuCl_2_ promoted differentiation to caulonema at the same concentration (Fig. 2E). These results suggest that an increase in environmental Cu concentration causes the differentiation of chloronema to caulonema in *S. cataractae*.

### Effects of various heavy metals on the differentiation of chloronema to caulonema

**Fig. 3.**
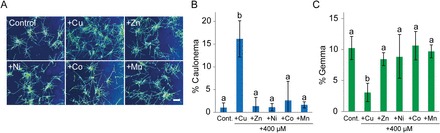
Effects of various heavy metals on cell differentiation from chloronema in *Scopelophila cataractae*. (A) Gemmae were germinated on BCDAT agar media containing 0.22 µM CuSO_4_ (Control), 400 µM CuSO_4_ (+Cu), 400 µM ZnSO_4_ (+Zn), 400 µM NiSO_4_ (+Ni), 400 µM CoSO_4_ (+Co), and 400 µM MnSO_4_ (+Mn). Photographs of growing protonema were obtained after 10 d of incubation. Scale bar=1mm. (B and C) Population percentages of caulonema cells (B) and gemmae (C) after 10 d of culture on agar media. Values represent the means±SD of three biological replicates. Different letters indicate statistically significant differences as detected by Tukey–Kramer tests (*P*<0.05) following ANOVA.

We previously showed that, in comparison to the model moss *P. patens*, *S. cataractae* is tolerant to several heavy metals such as Cu, zinc, cobalt, nickel, and silver (Nomura and Hasezawa, 2011). This finding raises the question whether these heavy metals also affect protonemal cell differentiation in *S. cataractae*. We tested the effects of 400 µM MnSO_4_, CoSO_4_, NiSO_4_, or ZnSO_4_ on *S. cataractae* cell growth and differentiation. These heavy metals did not lead to an increase in caulonema population or decrease in gemma population (Fig. 3A–C), suggesting that cell differentiation from chloronema to caulonema or gemma is specifically regulated by environmental Cu concentration.

### Effects of Cu on endogenous phytohormone contents

**Fig. 4.**
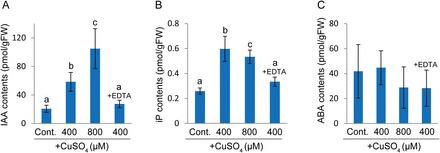
Endogenous levels of phytohormones in *Scopelophila cataractae* grown on media containing different copper concentrations. Gemmae were germinated on BCDAT agar media containing 0.22 (control), 400, or 800 µM CuSO_4_ or 400 µM CuSO_4_ and 400 µM EDTA. After 10 d of incubation, the growing protonemata were harvested, and the endogenous levels of IAA (A), N^6^-(*∆* ^2^-isopentenyl)adenine (iP) (B), and ABA (C) were quantified. Values represent the means±SD of six biological replicates. FW, fresh weight. Different letters indicate statistically significant differences as detected by Tukey–Kramer tests (*P*<0.05) following ANOVA.

The differentiation of chloronema to caulonema is positively regulated by auxin in several mosses (Cove *et al.*, 2006), implying that high Cu concentrations might affect endogenous auxin levels in *S. cataractae*. To test this hypothesis, we analysed the effect of Cu on endogenous phytohormone levels in protonemata cultured for 10 d. Endogenous IAA contents were 2.8- and 5-fold higher in *S. cataractae* grown in the presence of 400 and 800 µM CuSO_4_, respectively, than those grown under control conditions (Fig. 4A). Moreover, this Cu-induced IAA accumulation was repressed by co-incubation with EDTA (Fig. 4A), which repressed the Cu-induced differentiation of chloronema to caulonema (Fig. 2A–D). Besides IAA, accumulation of cytokinin N^6^-(Δ^2^-isopentenyl)adenine (iP) also increased after Cu treatment (Fig. 4B), whereas other active forms and their conjugates were not stably detected or their concentrations were not significantly altered upon Cu treatment (Supplementary Table S2). There was no significant difference in abscisic acid (ABA) content following any of the treatments (Fig. 4C). These results suggest that environmental Cu is involved in the regulation of IAA and iP accumulation in *S. cataractae*.

### Effects of auxin on protonemal cell differentiation

**Fig. 5.**
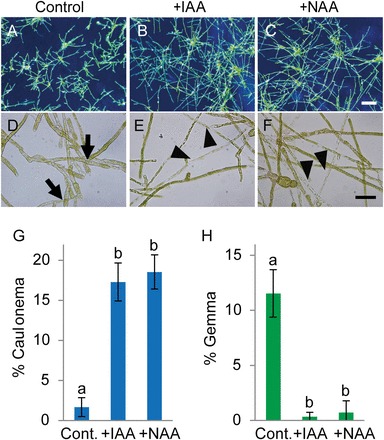
Effects of exogenously applied auxin on cell differentiation from chloronema in *Scopelophila cataractae*. Gemmae were germinated on BCDAT agar media containing 0.1% dimethyl sulfoxide (DMSO) (control; A, and D), 0.5 µM IAA and 0.1% DMSO (+IAA; B and E), or NAA and 0.1% DMSO (+NAA; C and F). The Cu concentration was 0.22 µM. Photographs of the growing protonema were obtained after 10 d of incubation. D–F are magnified images of protonemal cells from A–C, respectively. The arrows in D indicate gemmae formed at the protonema tips. The arrowheads in E and F indicate the caulonema cells. Scale bars=1mm for A–C, and 50 µm for D–F. The population percentages of caulonema cells (G) and gemmae (H) after 10 d of culture on agar media were quantified. Values represent the means±SD of four independent experiments. Different letters indicate statistically significant differences as detected by Tukey–Kramer tests (*P*<0.05) following ANOVA.

Previous studies suggested that auxin treatment might regulate the differentiation of chloronema to caulonema in several mosses (Cove *et al.*, 2006). Therefore, we investigated the effects of auxin application on cell differentiation. Even under low Cu conditions (control), application of 0.5 µM NAA or IAA promoted the differentiation of chloronema to caulonema (Fig. 5A–G), and protonemal gemma formation was greatly repressed by auxin treatment (Fig. 5H). This response to auxin is very similar to that elicited by high Cu conditions (Fig. 1D–I, K, L), suggesting that the Cu-regulated cell differentiation from chloronema to caulonema or gemma in *S. cataractae* is mediated by auxin. On the other hand, treatment with the cytokinin BAP had no effect on cell differentiation (Supplementary Fig. S3).

### Effects of an auxin antagonist and a biosynthesis inhibitor on the Cu-induced differentiation of chloronema to caulonema

**Fig. 6.**
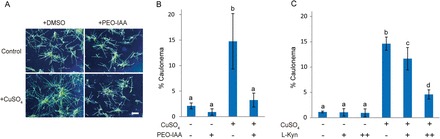
Effects of the auxin antagonist, PEO-IAA, and auxin biosynthesis inhibitor on the copper-induced differentiation of chloronema to caulonema in *Scopelophila cataractae*. (A) Gemmae were germinated on BCDAT agar media containing 0.22 µM (Control) or 400 µM CuSO_4_ (+CuSO_4_) and with 0.1% DMSO (+DMSO) or 20 µM PEO-IAA and 0.1% DMSO (+PEO-IAA). Photographs of the growing protonema were obtained after 10 d of incubation. Scale bar=1mm. (B) Population percentages of caulonema cells after 10 d of culture in conditions specified in A. Values represent the means±SD of four independent experiments. (C) Gemmae were germinated on BCDAT agar media containing 0.22 µM (CuSO_4_: –) or 400 µM CuSO_4_ (CuSO_4_: +) and with 0.05% DMSO or 20 µM (l-kyn: +) or 40 µM (l-kyn: ++) l-kynurenine and 0.05% dimethyl sulfoxide (DMSO). After 10 d of incubation, the population percentages of caulonema cells were quantified. Values represent the means±SD of four independent experiments. Different letters indicate statistically significant differences as detected by Tukey–Kramer tests (*P*<0.05), followed by ANOVA.

To investigate the role of auxin signalling in Cu-induced caulonema differentiation in *S. cataractae*, we used the auxin antagonist PEO-IAA, which inhibits TIR1/AFBs-mediated auxin signalling (Hayashi *et al.*, 2012). Auxin-induced differentiation was significantly repressed when *S. cataractae* was co-incubated with NAA and PEO-IAA (Supplementary Fig. S4), indicating an antagonistic effect of PEO-IAA on *S. cataractae*. We further investigated the effect of PEO-IAA on the Cu-dependent differentiation of chloronema to caulonema. The population percentage of Cu-induced caulonema cells was repressed from 14.8% to 3.2% by 20 µM PEO-IAA (Fig. 6). To further investigate the involvement of auxin in the Cu-dependent differentiation of chloronema to caulonema, we evaluated the effect of l-kynurenine, which is reported to be an inhibitor of the auxin biosynthesis enzyme TAA1/TAR in *Arabidopsis thaliana* (He *et al.*, 2011). Treatment with 20 or 40 µM l-kynurenine was found to dose-dependently repress the population percentage of caulonema after treatment with 400 µM CuSO_4_ (Fig. 6C). These results support the hypothesis that Cu-induced caulonema differentiation in *S. cataractae* is mediated by auxin signalling.

## Discussion

In nature, the typical copper moss, *S. cataractae*, is only found in Cu-rich environments, but how this species flourishes in such special habitats was not clear. Our results clarify the mechanism underlying the Cu-dependent exuberance of *S. cataractae*, an obligate metallophyte.

In *S. cataractae*, high Cu conditions did not affect gemma germination and promoted the differentiation of chloronema to caulonema (Fig. 1). On the other hand, low Cu conditions promoted asexual reproduction via the formation of protonemal gemmae (Fig. 1). These findings suggest that a high concentration of Cu is a favourable condition for the vegetative protonemal growth of *S. cataractae*. Intriguingly, although *S. cataractae* is tolerant to other heavy metals, only Cu induced the differentiation of chloronema to caulonema or gemma (Fig. 3) (Nomura and Hasezawa, 2011). This observation suggests that *S. cataractae* has a specific Cu-sensing mechanism that allows it to live in Cu-rich environments.

In other mosses, including *P. patens* and *F. hygrometrica*, differentiation of chloronema to caulonema is positively regulated by auxin. Our results suggest that *S. cataractae* employs a similar mechanism of auxin-regulated caulonema differentiation. In addition, protonemal gemma formation in *S. cataractae* was repressed by auxin treatment under low Cu conditions (Fig. 5). That is, auxin treatment elicited similar effects as those induced under high-Cu conditions. Notably, treatment with high concentrations of Cu increased the endogenous IAA concentration (Fig. 4), and the auxin antagonist or auxin biosynthesis inhibitor l-kynurenine inhibited Cu-dependent differentiation of chloronema to caulonema (Fig. 6). These findings suggest that high concentrations of Cu are not essential for nutrition; rather, they induce auxin accumulation and promote caulonema cell differentiation. In fact, we were able to maintain *S. cataractae* protonema in liquid cultures in normal BCDAT medium at the basal Cu concentration (0.22 µM), although these culture became gemmae rich.

The molecular mechanisms underlying the Cu-dependent IAA accumulation in *S. cataractae* remain unknown. Genes encoding homologues of *Arabidopsis* SHI/STY family proteins, which are positive regulators of the IAA biosynthesis gene *YUCCA4* (Eklund *et al.*, 2010*a*; Mashiguchi *et al.*, 2011), regulate caulonema differentiation via auxin synthesis in *P. patens* (Eklund *et al.*, 2010*b*). On the other hand, studies in *Arabidopsis* have shown that the PHYTOCHROME-INTERACTING FACTOR family of bHLH transcription factors acts as a growth regulator through auxin production via the regulation of YUCCA expression in response to various environmental conditions such as light, soluble sugar content, and temperature (Franklin *et al.*, 2011; Lilley *et al.*, 2012; Ljung, 2013; Nomoto *et al.*, 2012; Sun *et al.*, 2013). Therefore, the environmental Cu level was thought to regulate auxin production, which is mediated by the above-described mechanisms in *S. cataractae*. In addition, it was speculated that subsequent auxin signalling might control protonemal cell differentiation.

Our phytohormone quantification revealed that a cytokinin, iP, accumulated under high-Cu conditions (Fig. 4B). However, cytokinins were not thought to play a major role in initiating the differentiation of chloronema to caulonema and protonemal gemma formation in *S. cataractae* because there were no visible effects of BAP treatment on cell differentiation (Supplementary Fig. S3), and other cytokinins and conjugates were not significantly affected (Supplementary Table S2). The co-existence of cytokinin and auxin was shown to induce bud formation in *P. patens* (Aoyama *et al.*, 2012; Ashton *et al.*, 1979; Cove *et al.*, 2006; Decker *et al.*, 2006; Schumaker and Dietrich, 1998). Thus, cytokinins might facilitate bud formation in the later vegetative developmental stage in *S. cataractae*.

How *S. cataractae* tolerates heavy metals is not fully understood. A previous study reported that cell wall pectin is the major facilitator of Cu accumulation in *S. cataractae* (Konno *et al.*, 2010). Because the species is also tolerant to other heavy metals, multiple mechanisms might be involved in the hyper-tolerance. Recent studies have revealed several molecular mechanisms underlying plant heavy metal tolerance, including chelation, transportation, and sequestration systems (Cobbett, 2000; Hall, 2002; Hall and Williams, 2003; Haydon and Cobbett, 2007; Krämer *et al.*, 2007; Rascio and Navari-Izzo, 2011). Further investigation is necessary to completely understand the heavy metal tolerance capacities of *S. cataractae*.

**Fig. 7.**
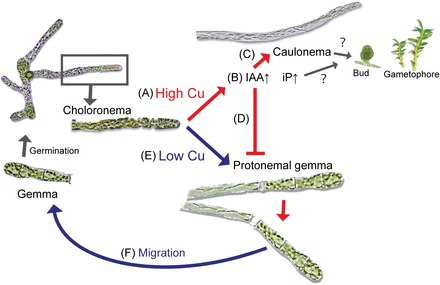
Hypothetical model of *Scopelophila cataractae* habitat expansion into Cu-rich environments. In Cu-rich conditions (A), chloronema germinates from gemma and accumulates IAA (B). Accumulation of IAA promotes transition of chloronema to caulonema (C) and represses protonemal gemma formation via its signalling pathway (D). Germinated chloronema promotes protonemal gemma formation in low-Cu environments (E). Gemmae released from protonema then migrate to new locations (F).

In conclusion, the results of this study suggest that the copper moss *S. cataractae* has unique physiological and developmental features as an obligate metallophyte, and these features might explain the propagation of *S. cataractae* in Cu-rich environments (Fig. 7). *S. cataractae* might have acquired the mechanisms of Cu-dependent phytohormone accumulation and cell differentiation during the evolutionary process to benefit from its advantageous Cu-tolerance ability. Further studies are needed to elucidate the mechanism underlying this tolerance to Cu and to understand the evolution of copper mosses and other metallophytes.

## Supplementary data

Supplementary data can be found at JXB online.

Figure S1. Gemmae collection and culture methods.

Figure S2. Chloronema, protonemal gemma, and caulonema in *Scopelophila cataractae*.

Figure S3. Effects of cytokinin on *S. cataractae* protonemal differentiation.

Figure S4. Effects of the auxin antagonist PEO-IAA on auxin-induced caulonema differentiation in *S. cataractae*.

Table S1. Soluble copper contents in soil extracts of *S. cataractae* habitat.

Table S2. Endogenous levels of cytokinins and conjugates in *S. cataractae* grown on media with different copper concentrations.

Supplementary Data
