# Supplementary material for: Copper mediates auxin signalling to control cell differentiation in the copper moss Scopelophila cataractae
Source: J Exp Bot. 2014 Nov 26;66(5):1205–13. doi: 10.1093/jxb/eru470 (PMC4339587; doi:10.1093/jxb/eru470)
Supplement: Supplementary Data [file supp_66_5_1205__index.html]

Copper mediates auxin signalling to control cell differentiation in the copper moss Scopelophila cataractae — Copper mediates auxin signalling to control cell differentiation in the copper moss Scopelophila cataractae — Copper mediates auxin signalling to control cell differentiation in the copper moss Scopelophila cataractae — Supplementary Data 

# Copper mediates auxin signalling to control cell differentiation in the copper moss *Scopelophila cataractae*

## Supplementary Data

Data files

**Files in this Data Supplement:**

- Supplementary Data - Supplementary Data
