# Supplementary material for: Copper mediates auxin signalling to control cell differentiation in the copper moss Scopelophila cataractae
Source: J Exp Bot. 2014 Nov 26;66(5):1205–13. doi: 10.1093/jxb/eru470 (PMC4339587; doi:10.1093/jxb/eru470)
Supplement: Supplementary Data [file supp_eru470_jexbot137240_file001.pdf]

Supplementary Data

Copper mediates auxin signaling to control cell differentiation in the copper moss *Scopelophila cataractae*

Toshihisa Nomura[1,2], Misao Itouga[1], Mikiko Kojima[1], Yukari Kato[1], Hitoshi Sakakibara[1], Seiichiro Hasezawa[2,3]

[1]RIKEN Center for Sustainable Resource Science, Tsurumi, Yokohama 230-0045, Japan

[2]Department of Integrated Biosciences, Graduate School of Frontier Sciences, The University of Tokyo, Kashiwanoha, Kashiwa, Chiba, Japan

[3]Advanced Measurement and Analysis, Japan Science and Technology Agency, Chiyoda-ku, Tokyo, Japan

E-mail addresses of the authors: Toshihisa Nomura, toshihisa.nomura@riken.jp; Misao Itouga, misao.itouga@riken.jp; Mikiko Kojima, mikiko@postman.riken.jp; Yukari Kato, yukari.kato@riken.jp; Hitoshi Sakakibara, sakaki@postman.riken.go.jp; Seiichiro Hasezawa, hasezawa@k.u-tokyo.ac.jp

Corresponding author: Toshihisa Nomura, RIKEN Center for Sustainable Resource Science, Tsurumi, Yokohama 230-0045, Japan

E-mail: toshihisa.nomura@riken.jp, Tel: +81-45-503-9576, FAX: +81-45-503-9609

Supplementary Fig. S1

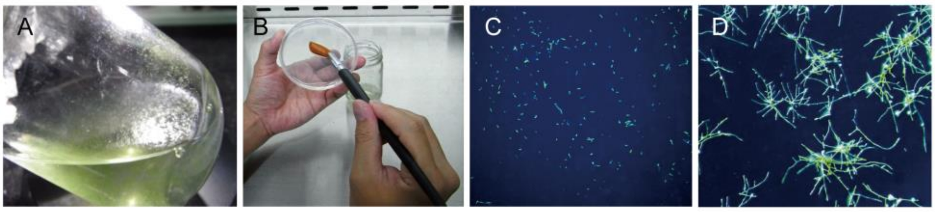

Supplementary Fig. S1

Gemmae collection and culture methods used in this study. (A) The gemmae adhered to and accumulated on the inner wall of the glass bottle. (B) The collected gemmae were resuspended in new BCDAT liquid medium and then spread on agar medium using an autoclaved paintbrush. (C, D) The gemmae on the BCDAT agar medium were cultured for 0 (C) or 10 (D) days.

Supplementary Fig. S2

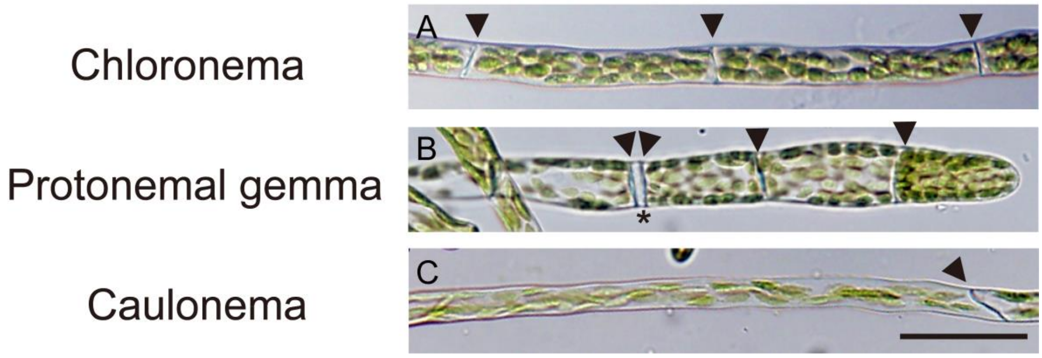

Supplementary Fig. S2

Chloronema (A), protonemal gemma (B), and caulonema (C) in *Scopelophila cataractae*. The arrowhead indicates the cell plate, and the asterisk indicates the tmema cell before cell death. Scale bar = 30 µm.

Supplementary Fig. S3

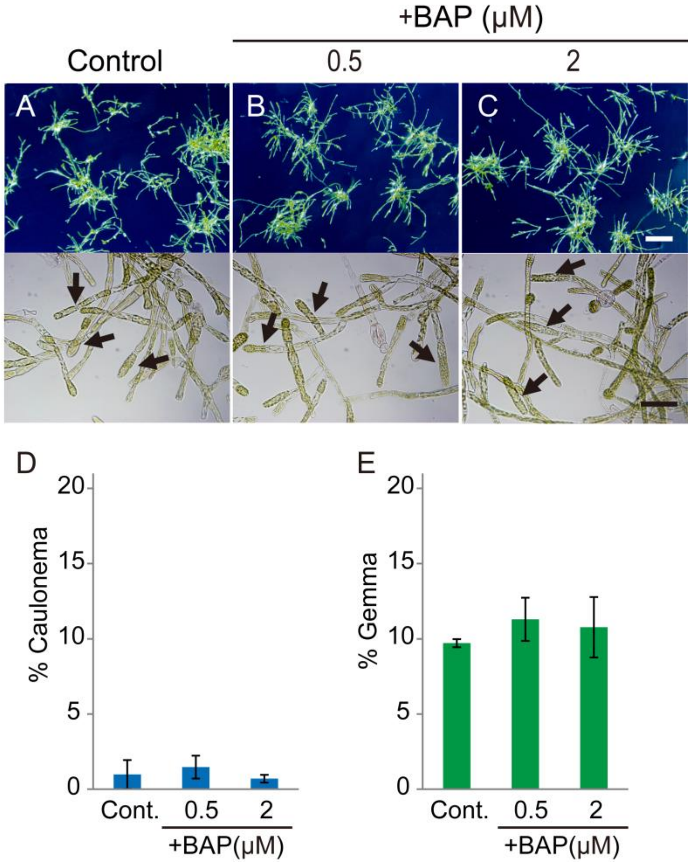

Supplementary Fig. S3

Effects of cytokinin on *Scopelophila cataractae* protonemal differentiation. *S. cataractae* protonema after 10 days of culture on agar medium containing DMSO (control) (A) or 0.5 (B) or 2 μM (C) BAP. Scale bar = 1 mm. Lower panels: magnified images of protonemal cells. The arrows indicate the gemma formed at the protonema tips and released gemma. Scale bar = 50 μm. Each picture represents a typical example. Quantification of the percentage of caulonema cells (D) and gemmae (E) after 10 days of culture on agar medium containing DMSO (control) or 0.5 or 2 μM BAP. Values represent the means ± SD of three independent experiments.

Supplementary Fig. S4

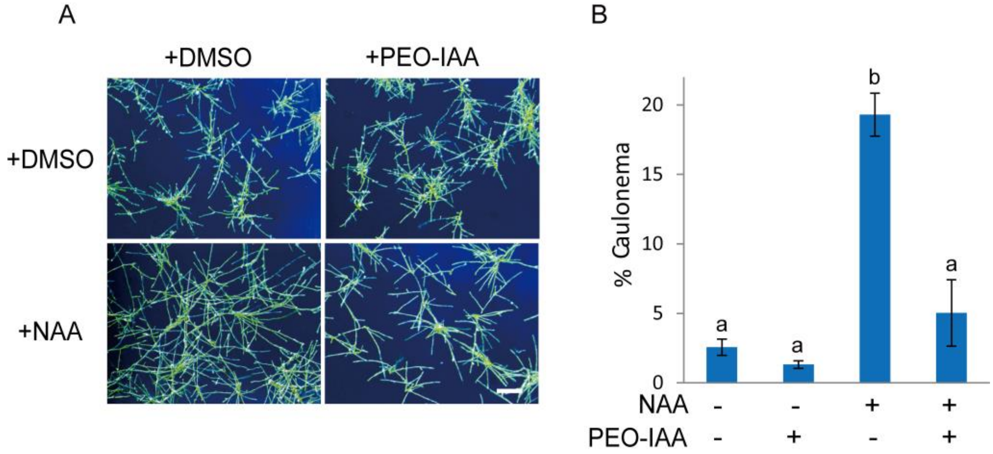

Supplementary Fig. S4

Effects of the auxin antagonist, PEO-IAA, on auxin-induced caulonema differentiation in *Scopelophila cataractae*. (A) *S. cataractae* protonema after 10 days of culture on agar medium containing DMSO or 0.5 μM NAA with DMSO or 20 μM PEO-IAA. Scale bar = 1 mm. (B) Quantification of the percentage of caulonema cells after 10 days culture on agar medium containing DMSO or 0.5 μM NAA with DMSO or 20 μM PEO-IAA. Values represent the means ± SD of three independent experiments. Different letters indicate statistically significant differences as detected by Tukey-Kramer tests ($P < 0.05$) following (ANOVA).

Supplementary Table 1

Sampling place	^{63}Cu conc. of soil extract (μM, n=3, ± SD)	Soil moisture content
Zenpukuji Temple -point1	1668.5 ± 55.5	58 %
Zenpukuji Temple -point2	787.4 ± 23.3	64 %
Zenpukuji Temple -point3	1709 ± 189.1	57 %
Zenpukuji Temple -point4	566.3 ± 70.7	60 %
Zenpukuji Temple -point5	161 ± 40.3	55 %
Tsurumi Shrine	807.1 ± 50.1	35 %

Supplementary Table S1

Copper concentration in the soil extracts of *Scopelophila cataractae* habitats. ^{63}Cu concentration of soil solution extracted from *S. cataractae* habitats in the Zenpukuji Temple, Ibaraki Prefecture and Tsurumi Shrine, Kanagawa Prefecture was analyzed by inductively coupled plasma mass spectrometry (ICP-MS).

Supplementary Table 2

	+$CuSO_4$ (μM)			
	Control	400	800	400 + EDTA
tZ	N.D.	N.D.	N.D.	N.D.
tZR	0.03 ± 0.01	0.04 ± 0.01	0.05 ± 0.02	0.04 ± 0.01
cZ	N.D.	N.D.	N.D.	N.D.
cZR	0.34 ± 0.08	0.38 ± 0.03	0.48 ± 0.1	0.40 ± 0.08
DZ	N.D.	N.D.	N.D.	N.D.
DZR	N.D.	N.D.	N.D.	N.D.
iP	0.26 ± 0.03	0.60 ± 0.1	0.53 ± 0.05	0.4 ± 0.08
iPR	0.03 ± 0.01	0.03 ± 0.01	0.04 ± 0.02	0.04 ± 0.02
tZ7G	N.D.	N.D.	N.D.	N.D.
tZ9G	N.D.	N.D.	N.D.	N.D.
tZOG	N.D.	N.D.	N.D.	N.D.
cZOG	2.47 ± 0.51	1.70 ± 0.51	1.64 ± 0.45	1.43 ± 0.25
tZROG	0.43 ± 0.07	0.39 ± 0.05	0.26 ± 0.04	0.40 ± 0.06
cZROG	20.72 ± 2.09	16.05 ± 2.18	13.79 ± 2.44	16.60 ± 2.29
DZ9G	N.D.	N.D.	N.D.	N.D.
iP7G	N.D.	N.D.	N.D.	N.D.
iP9G	N.D.	N.D.	N.D.	N.D.

pmol/gFW

Supplementary Table S2

Endogenous levels of cytokinins and conjugates in *Scopelophila cataractae* grown on different copper concentrations. Gemmae were germinated on BCDAT agar media containing 0.22 (control), 400, or 800 μM $CuSO_4$ or and 400 μM $CuSO_4$ and 400 μM EDTA. After 10 days of incubation, the growing protonemata were harvested, and the endogenous levels of cytokinins and conjugates were quantified. Values represent the means ± SD of at least five biological replicates. FW, fresh weight. N.D., not detected; cZ, *cis*-zeatin; tZ, *trans*-zeatin; iP, N6-(Δ2-isopentenyl)adenine; DZ, dihydrozeatin; cZR, cZ-riboside; tZR, tZ-riboside; iPR, iP-riboside; DZR, DZ-riboside; tZ7G, tZ-7-N-glucoside; iP7G, iP-7-N-glucoside; tZ9G, tZ-9-N-glucoside; iP9G, iP-9-N-glucoside; DZ9G, DZ-9-N-glucoside; cZOG, cZ-O-glucoside; tZOG, tZ-O-glucoside; cZROG, cZR-O-glucoside; tZROG, tZR-O-glucoside.
